# The multidimensional significance of virus-host protein interactions and their implications in the antiviral defense of plants

**DOI:** 10.1007/s44154-026-00309-1

**Published:** 2026-05-14

**Authors:** Juan Zhang, Chunyan Qi, Zengxu Wu, Yuyu Zhang, Jiangbo Shi, Zejun Li, Jun Guo, Xiangping Zhou, Shen Tang, Jian Yang, Peng Liu

**Affiliations:** 1 State Key Laboratory for Quality and Safety of Agro-Products, Key Laboratory of Biotechnology in Plant Protection of MARA, Key Laboratory of Green Plant Protection of Zhejiang Province, Institute of Plant Virology, Ningbo University, Ningbo, 315211 China; 2 Yongzhou Tobacco Company of Hunan Province, Yongzhou, Hunan 425000 China

**Keywords:** Virus–host interactions, Antiviral defense, Virus resistance, Protein‒protein interaction

## Abstract

Plant viruses pose a serious threat to the production of numerous economically valuable food and fuel crops worldwide. Exploring protein interaction networks between viruses and their hosts offers vital insights into the viral infection process and facilitates the development of innovative antiviral strategies for plants. In this review, we aimed to explore commonly used identification methods for protein‒protein interactions (PPIs) and the latest relevant advances, focusing on the advantages and limitations of various PPI identification methods, as well as the role of protein interaction networks involved in virus‒host interactions. This overview of plant‒virus interactions can significantly contribute to insights into viral pathogenesis and innovative strategies for plant antiviral defense.

## Introduction

Plant viruses are a group of intracellular pathogens that pose a serious threat to global food security (Wu et al. 2024). Annual economic losses caused by plant viral diseases are estimated to reach billions of dollars worldwide. Climate change and intensive monoculture exacerbate this crisis by accelerating viral evolution and host adaptation. Given that viruses replicate exclusively within living host cells, conventional chemical treatments are largely ineffective. Consequently, the genetic improvement of virus resistance in crops is essential for achieving sustainable agriculture. Analyzing the interaction networks between viruses and host proteins enables the precise identification of key genetic targets, providing crucial research resources for antiviral resistance breeding.

The core of viral infection lies in the intricate protein‒protein interaction (PPI) network between the virus and host proteins (Khan et al. 2011). As obligate intracellular parasites, viruses are considerably smaller than fungal and bacterial cells and depend extensively on interactions with host proteins to accomplish entry, replication, assembly, and progeny virion release (Shah et al. 2022). Furthermore, virus‒host PPIs serve as critical molecular interfaces linking viral infection to plant defense responses. A prominent example is their intersection with phytohormone signaling networks, which play central roles in the “arms race” between plants and pathogens under biotic stress (Berens et al. 2017; Sharma and Prasad 2025). Numerous viral proteins have been shown to physically interact with key components of the salicylic acid (SA), jasmonic acid (JA), and abscisic acid (ABA) signaling pathways, often suppressing host immunity to promote successful infection. Conversely, plant hosts can exploit specific PPIs as molecular cues to activate immune signaling and restrict viral spread. These bidirectional interactions highlight the dual roles of PPIs as both viral virulence factors and host defense regulators. Therefore, determining the interactions between viruses and host proteins is essential for a comprehensive understanding of viral pathogenesis and the development of effective preventive strategies against viral infections (Hochholdinger et al. 2006).

In recent years, various biophysical, biochemical, and bioinformatic techniques have been successfully used to predict and detect PPIs between viral and host proteins. Classic methods, such as yeast two-hybrid (Y2H), affinity purification coupled with mass spectrometry (AP-MS), and fluorescence resonance energy transfer (FRET), have provided invaluable insights into virus‒host interaction landscapes. However, each approach has distinct strengths and limitations in detecting interactions (Lalonde et al. 2008). Plant–virus interactions are mediated by complex protein networks in both parallel and sequential pathways, resulting in a broad spectrum of dynamic physiological outcomes. The single-type approach is insufficient to comprehensively capture this complexity. Consequently, reliance on any single experimental approach is insufficient to capture the full complexity of these interaction networks. Hence, adopting an integrative, dynamic, and network-centric research strategy is crucial for clarifying these multifaceted interactions.

Concomitant advances in proteomics and bioinformatics have generated extensive datasets encompassing protein sequences, structures, functions, and interaction information, providing a critical foundation for such integrative analyses (Gelman et al. 2021; Whisstock and Lesk 2003). These data have been systematically curated into publicly accessible protein interaction resources, including STRING and Pfam, which enables the visualization and interrogation of interaction networks across diverse biological contexts (Bateman et al. 2000; Christian et al. 2003; Damian et al. 2011; Punta et al. 2012). Beyond interaction prediction, these platforms offer visual depictions of protein networks, which facilitate a more nuanced understanding of protein linkages, empowering researchers to delve into potentially important proteins and biological pathways.

In this review, we first provide an overview of commonly used and emerging methods used to investigate virus‒host PPIs, with a critical evaluation of their strengths, limitations, and applicability to plant‒virus systems. We then summarize recent advances in mapping virus‒host PPI networks and discuss how these interactions shape key processes of viral pathogenesis and host immune responses. Building on these insights, we examine the molecular interplay between plant viruses and host proteins and its implications for the breeding of virus-resistant crops. Finally, we outline how knowledge of virus‒host PPIs can be leveraged to identify critical host factors and inform the rational development of virus-resistant crop varieties. Overall, this review provides a framework for exploiting protein interaction networks to increase antiviral resistance in plants.

## Current techniques for analyzing virus‒host protein interactions

In recent years, several experimental methods have been employed to predict and identify interactions between viruses and host proteins, revealing various types of interactions. This section summarizes several classical and contemporary techniques for studying protein‒protein interactions (PPIs) and the key features and limitations of each to help select effective methods.

### Common strategies offer identifying and characterizing protein interactions

**Fig. 1:**
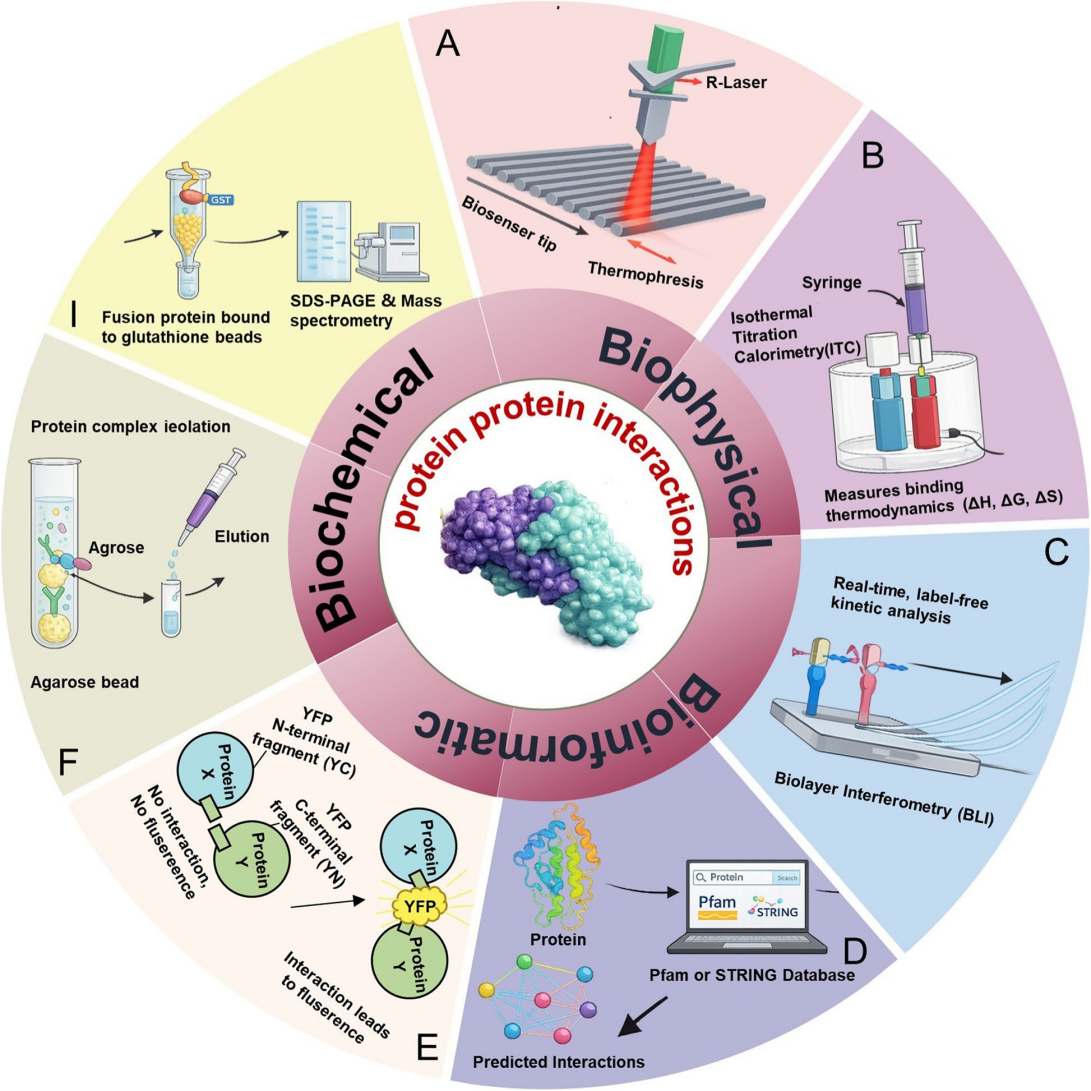
Current techniques for PPI detection. **a** Microscale thermophoresis (MST). The sample solution with fluorescent molecules in a capillary tube undergoes localized heating with an IR-laser. The thermophoretic movement of these fluorescent molecules across the temperature gradient is accurately monitored and measured. **b** Isothermal titration calorimetry (ITC). Both reference and sample chambers are set to the desired temperatures. Ligands is loaded into a precise injection system apparatus and introduced into the sample chamber cotaining the target protein. Sequential small-volume injections allow for the detection of ligand–protein interactions through measurable thermal shifts. **c** Bio-layer interferometry (BLI). The analyte solution contacts an optical biosensor with an immobilized ligand. Binding of analyte molecules changes the optical thickness at the sensor surface, causing a measurable shift in the interference pattern of reflected light. This real-time signal enables determination of binding kinetics and affinity without labeling. **d** STRING and Pfam. STRING is a database for analyzing protein–protein interaction networks by integrating experimental evidence, computational predictions, and literature data to reveal functional associations. Pfam is a protein domain database that identifies conserved domains and motifs within sequences using hidden Markov models, enabling functional and structural annotation. **e** Bimolecular Fluorescent Complimentary (BiFC). A fluorescent protein split into two non-active fragments, N and C, is fused to target proteins. Interaction brings these fragments together, reconstituting a functional fluorescent protein. **f** GST-pull down. The target protein is expressed as a fusion with GST and immobilized on glutathione (GSH) affinity resin, serving as a support with affinity for the target protein and acting as bait protein. The solution containing the target protein flows through the column, allowing the capture of interacting prey proteins. **g** Co-Immunoprecipitation (Co-IP). Bait-specific antibody capture bait–prey complex in the cell. Non-specific proteins bound to the target protein are washed away, and the remaining complexes are analyzed by SDS-PAGE and detected via Western blot

The classical methods for studying PPIs can be broadly divided into three primary categories: biophysical, biochemical, and bioinformatic methods (Fig. 1). Biophysical techniques are primarily used to detect changes in physical properties, such as kinetics or thermodynamics, associated with protein interactions. Biophysical techniques include isothermal titration calorimetry (ITC), microscale thermophoresis (MST), and biolayer interferometry (BLI).

MST is emerging as a sensitive detection and quantification method for biomolecular interaction analysis (Rainard et al. 2018), by monitoring changes in thermophoretic mobility and the local microenvironment of a fluorophore. When a labeled fluorescent protein is added to the ligand solution, the fluorescence intensity changes with the speed at which the labeled protein escapes from heat on the basis of binding at a certain concentration with the ligand molecules. MST can be used to establish a binding curve based on the variation in thermophoresis between the fluorescence of both unlabeled and labeled states against varying ligand concentrations. Additionally, MST enables the rapid and accurate identification and quantification of protein‒protein interactions (Khan et al. 2011; Magnez et al. 2022; Seidel et al. 2013) and allows the identification of direct interactions requiring purified proteins at very low sample concentrations (Jerabek-Willemsen et al. 2014). Therefore, MST offers significant advantages over other common methods for proteins that are difficult to express or inherently unstable. Another powerful method, ITC, involves measuring the heat changes induced by molecular interactions (Krell et al. 2014). An ITC experiment can directly reveal the comprehensive thermodynamic parameters associated with biomolecular interactions, such as enthalpy (ΔH), Gibbs free energy (ΔG), changes in entropy (ΔS), and binding affinity (Johnson 2021; Werber and Mastai 2018). In contrast to MST, ITC can be used to determine the receptor–ligand binding affinity in the natural state without fluorescence labeling. In addition to MST and ITC, BLI has emerged as a powerful label-free biophysical technique for the real-time analysis of protein‒protein interactions (Bates et al. 2025). BLI measures binding-induced changes in the interference pattern of white light reflected from the tip of fiber-optic biosensors, allowing the direct observation of association and dissociation kinetics without labeling or the immobilization of one binding partner. For example, BLI has been successfully applied to quantify binding affinities between viral effectors and host receptors and to screen small molecules that target specific virus–host protein interactions (Strauch et al. 2017). Collectively, these three techniques require minimal sample volumes, offer short experimental times, ensure high reproducibility, and exhibit low false-positive rates, providing substantial advantages for detecting PPIs. In the context of plant–virus interactions, these techniques are particularly valuable for characterizing transient or low-affinity PPIs in complex cellular environments.

Among the plethora of biochemical methods, co immunoprecipitation (Co-IP) and GST pull-down are the two principal methods used to analyze stable or strong protein‒protein interactions rather than transient interactions. In these two analyses, the bait protein is purified and precipitated, using a bait-specific antibody, from the total protein extracted from cells, followed by western blotting or MS analysis (Ransone 1995). Co-IP can be used to examine protein interactions in their natural state in vivo, providing an accurate representation of protein‒protein interactions. Conversely, GST pull-down assays are designed to assess the direct interaction between bait and prey proteins in vitro; however, the inherent properties of fusion tags might cause nonspecific binding, potentially complicating the analysis. Bimolecular fluorescence complementation (BiFC) is a novel approach that combines bait and prey proteins into complementary fluorescent protein fragments of a fluorescent protein. Upon interaction of the proteins of interest, the nonfluorescent fragments come into proximity and reconstitute an intact fluorescent protein. Therefore, BiFC allows the direct observation of protein‒protein interactions in living cells (Tunc-Ozdemir et al. 2016). Accordingly, given their respective advantages in Co-IP, GST pull-down, and BiFC, the combined application of these complementary tools will provide critical evidence regarding the physiological relevance, subcellular context, and stability of protein complexes.

Bioinformatics enables the robust validation of PPIs. It offers computational tools and databases crucial for predicting, analyzing, and confirming interactions observed in experimental setups (Rabbani et al. 2018). Many PPIs are mediated through specific domains, which can be analyzed using Pfam (Bateman et al. 2000; Farooq et al. 2021; Punta et al. 2012) and STRING (Christian et al. 2003), two prominent tools that significantly contribute to the prediction of PPIs. Pfam is a comprehensive domain database that combines extensive protein domain data required to infer potential interactions between proteins. This methodology relies on the comparison of a target protein sequence with reference domains to identify interaction patterns and potentially associated domains (Finn et al. 2014). STRING involves a comprehensive approach with the integration of protein interaction information from various sources and includes experimental validation, literature reviews, and computational predictions; it can be used to construct intricate protein interaction networks, presenting a comprehensive overview of how proteins interact within a biological system (Damian et al. 2011). Collectively, these tools enhance the reliability of PPI prediction and provide valuable insights into the structural basis and biological context of protein interactions.

Although numerous methods have been developed to detect PPIs, each method is subject to limitations when applied to plant–virus interaction studies. Biophysical techniques such as MST and ITC provide quantitative measurements of binding affinity, but their requirements for purified proteins in vitro cannot fully recapitulate the complex cellular environment in which virus–host interactions occur. Biochemical approaches, including GST pull-down and Co-IP assays, often fail to capture transient or weak PPIs during viral infection. In vivo imaging techniques such as BiFC allow the visualization of PPIs in living cells. However, BiFC is highly temperature sensitive. At higher temperatures, fluorescent protein fragments often fail to efficiently reassemble into a functional fluorophore, limiting the reliability under physiological conditions. Moreover, bioinformatics predictions depend heavily on existing datasets and domain annotations and therefore require experimental validation. The tug-of-war between plant hosts and viruses involves highly dynamic and multilayered interactions, and a reliance on any single approach is insufficient (Sharma et al. 2025). Accordingly, the integration of complementary strategies across in vitro, in vivo, and computational levels is essential for understanding dynamic virus‒host PPI networks.

### Advances in proteomic approaches for studying protein interactions

**Fig. 2:**
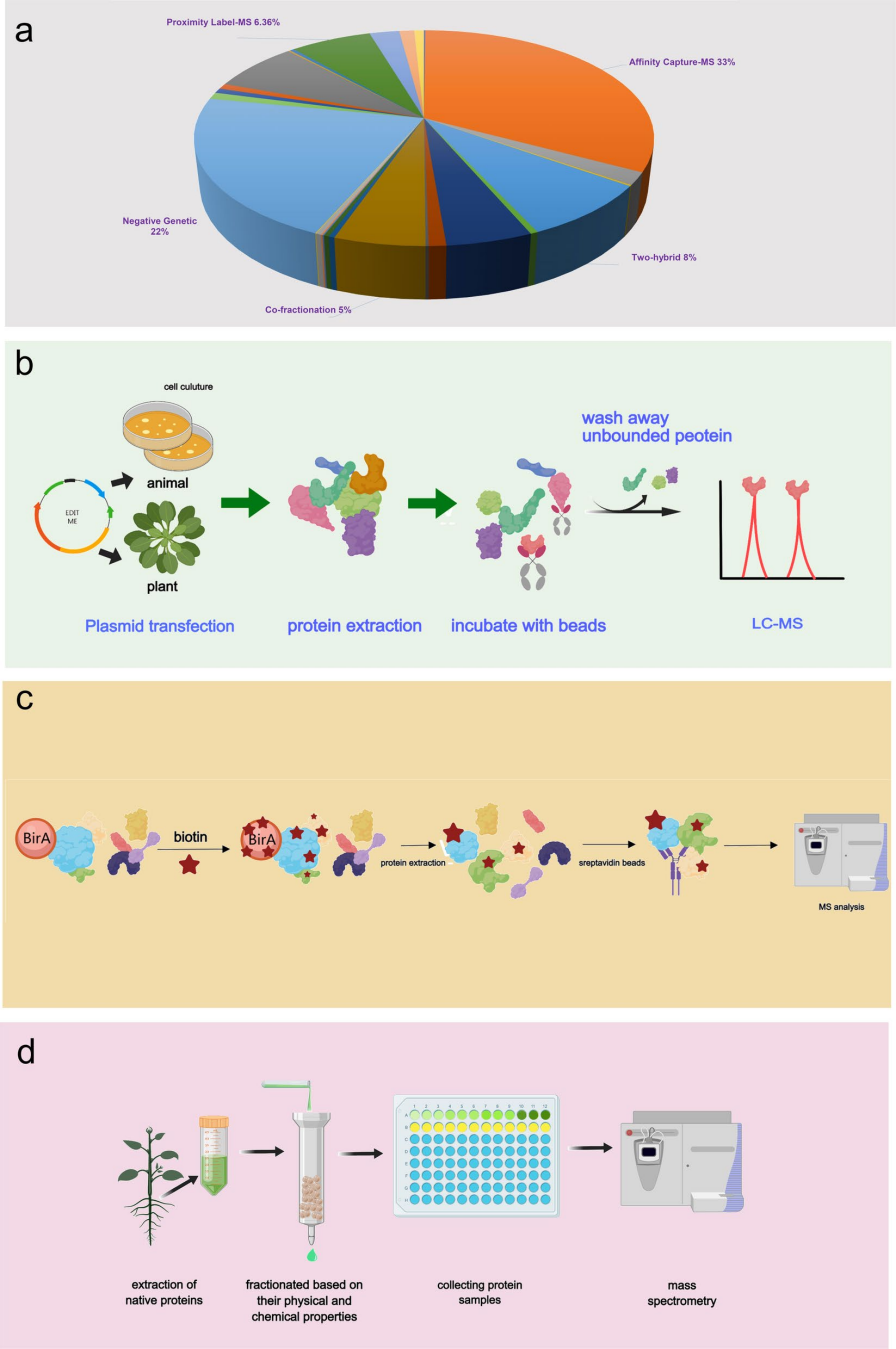
Illustration of some mass spectrometry‑based strategies for PPI detection. **a** The BioGRID database compiles protein interaction information derived from various experimental methods, among which studies based on mass spectrometry such as Affinity Capture-MS, Proximity Label-MS, and Co-fractionation collectively contribute to over 43% of the protein interaction data. **b** An illustrative overview of Affinity Purification-Mass Spectrometry (AP-MS) workflow, encompassing the initial plasmid transfection through to the culminating LC–MS profiling. **c** An illustrative overview of Proximity Label-MS techniques workflow. Initiated by the enzymatic biotinylation of a target protein via BirA ligase, selective affinity purification to isolate the biotin-conjugated protein. The procedure culminates in a comprehensive mass spectrometric analysis, delineating the proteomic landscape influenced by the proximity labeling. **d** An illustrative overview of Co-fractionation Mass Spectrometry (CF-MS) workflow. Proteins from plant tissues are isolated, chromatographically separated, and post-separation, analyzed via mass spectrometry to delineate protein identities and interactions

Proteomic approaches are being increasingly employed to elucidate virus‒host protein interactions at the molecular level. By the end of 2023, the Biogrid database, one of the largest protein interaction databases, had compiled more than 2.63 million protein interaction entries from 28 different experimental methods. Biogrid includes mass spectrometry-based techniques, such as affinity capture–MS, proximity label–MS, and co-fractionation, which collectively contribute more than 45% of the protein interaction data in the database (Fig. 2a). This trend underscores a growing preference for mass spectrometry in probing the intricacies of proteomic interactions, highlighting its pivotal role in advancing the molecular understanding of virus‒host dynamics.

AP‑MS is the predominant high-throughput method for studying PPIs (Low et al. 2021). This method relies on the selective affinity between specific antibodies, affinity reagents, and target proteins. Classic AP‒MS experiments usually include four key steps: (i) lysis of cells or tissues; (ii) incubation of the resultant protein mixture with antibody-conjugated affinity beads to capture the target protein complex; (iii) washing off nonspecifically bound and background proteins and eluting specific interacting proteins; and (iv) mass spectrometry detection and analysis of interacting proteins (Fig. 2b). However, AP‒MS has limited potential for detecting membrane protein interactions and effectively capturing transient and weak PPIs. To overcome these limitations, proximity-labeling MS techniques have been employed, which enhance the detection of transient and weak PPIs by facilitating the close association of target proteins with their biotinylated counterparts. Proximity labeling (PL), integrated with mass spectrometry, transcends the limitations of AP‒MS, enabling the comprehensive elucidation of PPIs within the dynamic milieu of living cells (Kang and Rhee 2022). BioID is among the most common applications (Roux et al. 2018); it involves the use of the biotin ligase BirA, which catalyzes the biotinylation of a target protein in the presence of biotin. Through streptavidin-mediated pull-down and mass spectrometry analyses, candidate protein-interacting molecules can be identified (Li et al. 2019; Roux et al. 2018) (Fig. 2c). However, the conventional biotinylation process, necessitating which requires 15–18 h for robust tagging, may not be ideal for certain applications. Recently, two novel ligases, TurboID and miniTurbo, were developed through the directed evolution of the BioID ligase. These enzymes can achieve substantial biotinylation within 10 min when incubated with an excess of biotin, significantly reducing experimental timelines and enhancing efficiency (Branon et al. 2017). Such advancements provide new tools for investigating protein interactions and functionalities, particularly in biological processes that require rapid and transient tagging. Moreover, the introduction of TurboID and miniTurbo has opened new avenues for the dynamic study of intracellular protein networks, facilitating the precise analysis of mechanisms underlying protein interactions under varying conditions and time points.

Leveraging the groundwork laid by proximity-labeling mass spectrometry approaches, cofractionation mass spectrometry (CF-MS) has considerably advanced the investigation of protein interactions (Fig. 2d). CF‒MS is a high-throughput method for detecting protein‒protein interactions and is applicable to various sample types (Fossati et al. 2020; Mcwhite et al. 2020); it involves the chromatographic separation of native protein extracts followed by identification of proteins within each biochemical fraction through MS. The phenomenon of protein coelution during this separation phase reflects protein interactions. In parallel, chemical cross-linking coupled with mass spectrometry (CXMS) combines chemical cross-linking with mass spectrometry (Tan et al. 2016). CXMS enables the capture of direct physical contacts between interacting proteins by covalently linking proximal amino acid residues, thereby providing distance constraints and structural insights into protein complexes in near-native states. This method is particularly important for analyzing transient or weak interactions. Beyond conventional MS and proximity labeling, emerging strategies further expand the detectable interactome and improve the preservation of labile complexes. Eyckerman et al. developed Virotrap, a lysis‑free method that incorporates a bait protein into virus-like particles to capture associated host factors under near-native conditions (Eyckerman et al. 2016). This approach minimizes purification-induced artifacts and allows both the detection of known binary interactions and the MS-based identification of novel protein partners.

In summary, advances in proteomics technologies have significantly expanded the understanding of the molecular landscape of plant–virus interactions. Integrating multiple proteomics approaches with traditional molecular biology validation methods (e.g., Y2H, BiFC or LCI) has become essential for reliable PPI analysis in plant stress biology. This multidimensional and integrative framework not only enhances the reliability of PPI data and eliminates false positives but also enables a more comprehensive understanding of how viruses exploit host vulnerabilities. In the future, with the increasing sensitivity of mass spectrometry and the evolution of single-cell proteomics, we will be able to more precisely identify key factors of viral resistance and susceptibility. These findings provide more targets for enhancing crop survival under viral stress through molecular design.

### Challenges and limitations in studying complex protein interaction networks

Protein interaction networks present fundamental regulators of most biological processes and the coordination of associated signal transduction, gene replication, transcription, translation, and cellular energy metabolism. As previously mentioned, numerous in vivo and in vitro PPI detection methods have been developed, each of which has unique detection profiles and limitations. There are two primary MS-related challenges in protein interaction research. First, this technology generates vast and complex datasets, demanding high data processing capabilities and greater depth and breadth of analysis. Second, the variability in protein abundance within samples complicates the detection of low-abundance proteins, posing a significant challenge in MS analysis (Low et al. 2021). Moreover, to understand the dynamic interactions and regulatory mechanisms among proteins, the development of more refined MS techniques and analytical methods that can accurately capture the subtle nuances within these complex biological processes is urgently needed.

PPIs are inherently transient and dynamic, which poses a considerable challenge in elucidating the conditions and mechanisms underlying protein interactions, especially when they are fleeting and weak. This intricacy highlights the imperative for researchers to move beyond the confines of single-method approaches for the validation of such interactions. This underscores the necessity of adopting a multidisciplinary strategy that integrates various techniques to achieve a comprehensive and rigorous understanding of PPIs within living cells.

Recent advances in artificial intelligence technologies have drawn significant attention to structure-based PPI prediction methods with the emergence of tools such as AlphaFold and RoseTTAFold, which have accelerated methodological progress in the discovery of protein interactions (Bryant et al. 2021; Jumper et al. 2021; Liu et al. 2022a, b; Ruff and Pappu 2021). Although structure-based approaches generally outperform sequence-based methods in terms of prediction accuracy, they often have a substantial computational burden. The complexity of protein structure modeling limits the scalability of these methods for large-scale PPI predictions. Furthermore, most current AI algorithms construct protein structures through training and learning based on known protein structures and gene evolutionary relationships, where information on gene evolution is derived primarily from multiple sequence alignments (MSAs) of existing gene sequences (Zhang et al. 2020a, b, c). Consequently, the actual predictive accuracy of these AI algorithms significantly depends on the quality of MSAs for the target proteins, which perform suboptimally on orphan proteins and protein complexes with limited homologous sequences. Further investigation and development of high-performance computational algorithms based on multidimensional, multiscale, and multimodal perspectives can elucidate potential interactive relationships within increasingly vast networks of protein‒protein interactions.

## Multidimensional significance of virus‒host protein interactions

Plant viruses are obligate parasites and have compact genomes that encode a limited number of proteins, typically ranging from a few to several dozen (Luan 2010; Sanfaçon 2015). The viral life cycle, which includes replication, assembly, and intrahost movement, is profoundly dependent on the host organism (Wang 2015). Infection with such viruses substantially alters protein expression in host plants; proteomic technologies enable the screening of these alterations and the identification of host proteins that interact with viral proteins, which not only aids in revealing how plants counteract viral infections through antiviral responses and how viruses deploy counterdefense strategies but also provides a theoretical basis for developing crop improvement techniques to increase plant resistance to viruses.

### The significance of protein interactions in viral pathogenesis and symptom development

During infections, viruses enter cells either through mechanical injury or are transmitted via vectors, after which intracellular replication is initiated. Following initial replication, viruses navigate through the plasmodesmata to invade neighboring cells, reach the vascular system, and spread to plant storage tissues via the phloem, resulting in systemic spread. This replication and movement cycle enables the viruses to establish widespread infection. Throughout this process, viruses strategically manipulate host proteins to increase infection efficiency and mitigate host immune responses, which has been reported across susceptible and resistant host plants (Di Carli et al. 2012). Accordingly, virus‒host PPIs operate at multiple stages of the viral life cycle and converge on distinct antiviral pathways and cellular processes. The interactions between virus and host proteins are summarized in Table 1 and are further discussed below.

**Table 1:** Summary of interactions between viral and host proteins and their impact on host defense

Virus Name	Virus protein	Target host protein	Function
TCV	CP	SGS3	Increases VSR activity and viral infection ability
PLRV	P0	AGO1	Promotes AGO1 degradation via autophagy to suppress defense
BBSV	CP	14–3-3a	Suppresses MAPKKKα-mediated antiviral innate immune response
CLCuMuV	βC1	MPK4	Inhibits MAPK-mediated defense and promotes symptoms
TuMV	NIb	NPR1	Suppresses salicylic acid-mediated antiviral signaling pathway
RDV	P2	OsIAA10	Affects the auxin pathway and transcription of downstream genes
CWMV	CP	GAPC	Disrupts host cell autophagic antiviral mechanisms
BSMV	γb	ATG7	Perturbs ATG12-ATG5 and ATG7-ATG3 conjugation systems
TuMV	6K2	NBR1	Impairs autophagic flux to reduce the degradation of HCpro proteins
TMV	CP	IP-L	Suppresses NbCML30 expression to inhibit downstream immune defense
TEV	VPg	eIF4E	Recruits translation factors for cap-independent viral translation
LMV	VPg	eIF4E	Recruits translation factors and 40S ribosomal subunits to viral RNA
PVMV	VPg	eIF(iso)4E	Utilizes specific eIF4E family members to enable host-specific infection
TBSV	p33	HSP70	Facilitates accumulation on peroxisomal membranes and assembly of the VRC
AbMV	MP	HSP70	Accumulates at cell periphery and chloroplasts to aid cell-to-cell movement
TSWV	MP	Sw-5b	Recognizes the NSm21 peptide to confer broad-spectrum resistance

One major target of virus–host PPIs is RNA-based immunity, which operates at multiple regulatory layers. RNA silencing is a central antiviral defense mechanism in plants, and many viruses encode viral suppressors of RNA silencing (VSRs) to counteract this pathway (Li and Wang 2019). Several viruses directly interfere with the core machinery of post-transcriptional gene silencing (PTGS). For example, turnip crinkle virus (TCV; *Betacarmovirus brassicae*) encodes a 38 kDa CP protein that interacts with SGS3 to further increase its VSR activity and infection ability (Liu et al. 2023a, b). Additionally, the p19 protein of tomato bushy stunt virus (TBSV; *Tombusvirus lycopersici*) functions as a potent VSR by binding to small interfering RNAs (siRNAs), preventing their incorporation into the RNA-induced silencing complex (RISC) and thereby inhibiting antiviral RNA degradation (Qiu et al. 2002). Likewise, the F-box protein P0 of potato leafroll virus (PLRV; *Polerovirus* PLRV) suppresses antiviral defense by promoting the autophagic degradation of ARGONAUTE1 (AGO1), a core component of the RNA silencing machinery (Derrien et al. 2012). Beyond the classical PTGS, recent studies indicate that viruses can also target RNA-directed DNA methylation (RdDM). During rice grassy stunt virus (RGSV; *Tenuivirus oryzabrevis*) infection, the viral P3 protein promotes P3IP1-dependent degradation of NRPD1a, thereby suppressing RdDM-associated antiviral defense (Zhang et al. 2020a, b, c). Recent studies further revealed that this process is also regulated by the upstream kinase SERK4. Specifically, P3 induces SERK4 expression, and SERK4-mediated phosphorylation of P3IP1 further promotes NRPD1a degradation, ultimately facilitating viral infection (Wu et al. 2025). Viral suppression of host RNA-based immunity also extends to the level of microRNA biogenesis. A recent study showed that a plant bunyaviral protein disrupts the phase separation of SERRATE, a key factor in miRNA processing, thereby disrupts the assembly of dicing bodies (D-bodies) and significantly reducing miRNA biogenesis (Zou et al. 2025). Together, these findings indicate that plant viruses can attenuate host RNA-based immunity through multilayered mechanisms, including direct suppression of silencing components, disruption of RdDM, and perturbation of condensate-dependent RNA regulatory processes. This multilayered targeting strategy highlights RNA silencing as a central and highly vulnerable hub in antiviral defense.

In addition to RNA-based immunity, viruses frequently target host signaling networks that coordinate immune activation. MAPK cascades represent one such major target. For example, beet black scorch virus (BBSV; *Betanecrovirus betae*) CP proteins can suppress the MAPKKKα-mediated antiviral innate immune response through interactions with 14–3-3a in plant (Gao et al. 2022). Similarly, the βC1 protein of *geminiviruses*, such as cotton leaf curl Multan virus (CLCuMuV; *Begomovirus gossypimultanense*), suppresses MAPK-mediated defense by directly interacting with and inhibiting MKK2 and MPK4, promoting viral infection and symptom development (Hu et al. 2019). Phytohormone signaling is another major regulatory layer manipulated by viruses (Zhao and Li 2021). Viruses can affect plant immunity by disrupting the biosynthesis or signal transduction of these hormones (Chen and Ding 2020). SA signaling is a key pathway that mediates resistance to viral pathogens. The NIb protein of turnip mosaic virus (TuMV; *Potyvirus rapae*), which belongs to the genus Potyvirus, targets the salicylic acid receptor NPR1, preventing the interaction and modification of NPR1 with SUMO3. This blockade inhibits subsequent SUMOylation-dependent phosphorylation processes, thereby suppressing the salicylic acid-mediated plant antiviral signaling pathway, which facilitates viral infection (Liu et al. 2023a, b). In addition to SA, auxin signaling has been implicated in virus-induced symptom development. The rice dwarf virus (RDV; *Phytoreovirus alphaoryzae*) P2 capsid protein competitively binds to OsIAA10 with the auxin receptor OsTIR1, thereby inhibiting the degradation of the OsIAA10 protein through the 26S proteasome. This leads to the accumulation of the OsIAA10 protein, affecting the auxin pathway and the transcription of downstream genes, thus promoting RDV infection and the development of disease symptoms (Lian et al. 2016). Taken together, viral infection profoundly rewires host signaling pathways. Although plants activate multiple immune-related signaling cascades to restrict viral replication, movement, and symptom development, viruses have evolved effective counterdefense strategies that hijack key host components within these networks to facilitate infection and pathogenesis.

Viruses also interfere with other upstream signaling pathways, including calcium signaling and environmental light signaling. For example, The TMV CP protein can interact with IP-L and suppress the expression of NbCML30, a novel calmodulin-like protein, at the nucleic acid and protein levels, thereby affecting a series of downstream host immune defense responses and enhancing infection (Liu et al. 2022a, b). The RNA Dependent RNA Polymerases (RdRPs) of CMV, PVY, and TMV disrupt the phyA–FHY1 interaction, thereby blocking phyA nuclear transport and impairing far-red light-mediated immunity (Gong et al. 2025). Together, calcium signaling and light signaling represent important upstream regulatory pathways in plant antiviral immunity, playing key roles in integrating intracellular and environmental signals (Gong et al. 2025; Wang et al. 2021; Zhang et al. 2024; Zvereva et al. 2024;). By targeting these pathways, viruses can efficiently suppress multiple downstream defense outputs, thereby enhancing infection efficiency and pathogenicity.

Autophagy has emerged as a key cellular pathway that is frequently manipulated by plant viruses during infection (Huang et al. 2019). For example, Chinese wheat mosaic virus (CWMV; *Furovirus chinense*) utilizes a similar strategy: its CP directly interacts with the host protein GAPC, disrupting the host cell autophagic antiviral mechanism and promoting viral accumulation as well as pathogenic effects (Niu et al. 2022). The γb protein of barley stripe mosaic virus (BSMV; *Hordeivirus hordei*) interacts with the host autophagy-related protein ATG7 and competitively interferes with the ATG7–ATG8 interaction, thereby suppressing autophagy-mediated antiviral defense and promoting viral infection (Yang et al. 2018). Additionally, the host-selective autophagy receptor NBR1 recognizes and mediates the autophagic degradation of TuMV replication-associated proteins, such as HCpro, thereby restricting viral accumulation (Hafrén et al. 2018). To counteract this antiviral defense, the TuMV proteins VPg and 6K2 impair NBR1-dependent autophagic flux and reduce HCpro degradation, illustrating the dynamic antagonism between host antiviral autophagy and viral counterdefense. Taken together, these studies show that virus–host PPIs do not act in isolation, but instead converge on a limited number of regulatory hubs, including RNA metabolism, signaling cascades, hormone pathways, and protein quality-control systems. Through these multilayered interactions, viruses not only enhance replication and systemic spread, but also reshape host physiology, promote symptom development, and establish successful infection.

Owing to the complexity of virus–host interaction networks, efficient identification of protein–protein interactions has become essential for dissecting the molecular basis of viral pathogenesis and host defense. Advances in interaction-mapping approaches, including yeast two-hybrid screening, co-immunoprecipitation coupled with mass spectrometry, proximity labeling, and AI-assisted structural prediction, have greatly accelerated the discovery of key viral and host factors as well as the reconstruction of regulatory interaction modules associated with infection.

### Significance of protein interactions in viral replication and movement

In addition to suppressing host defense pathways, efficiently exploiting host cellular protein factors for genome replication and progeny production represents a fundamental challenge for viruses. To meet this requirement, plant viruses frequently recruit host proteins that are directly required for viral replication and movement. Potyvirus utilizes the VPg protein for cap-independent translation. The VPg protein covalently attaches to the 5’ end of viral RNA and interacts with eIF4E by mimicking the cap structure of eukaryotes, thereby recruiting translation initiation factors and the 40S ribosomal subunit to viral RNA rather than host cell mRNA (Khan et al. 2008). Notably, Potyvirus exhibits host-specific utilization of different members of the eIF4E family. For instance, tobacco etch virus (TEV; *Potyvirus nicotianainsculpentis*) and lettuce mosaic virus (LMV; *Potyvirus lactucae*) recruit eIF4E in pepper and lettuce, respectively, whereas eIF(iso)4E is required for efficient infection in *Arabidopsis* (Estevan et al. 2014; Nicaise et al. 2003; Ruffel et al. 2002). Similarly, pepper veinal mottle virus (PVMV; *Potyvirus capsivenae*) requires both eIF4E1 and eIF(iso)4E for infection in pepper but preferentially utilizes eIF4E2 in tomato (Moury et al. 2014; Ruffel et al. 2006).

Most viral infections depend on host chaperone proteins to ensure the proper folding and functional maturation of newly synthesized viral proteins. Among these proteins, heat shock protein 70 (HSP70), a highly conserved molecular chaperone in eukaryotes, is among the most frequently utilized host proteins by viruses (Berka et al. 2022; Singh et al. 2024; Yang et al. 2023). HSP70 regulates viral infection by facilitating protein folding and protein trafficking and modulating the assembly of protein complexes (Wu et al. 2023). The replication enzymes p33 and p92^pol^ of tomato bushy stunt virus (TBSV; *Tombusvirus lycopersici*) require HSP70 for their accumulation on peroxisomal membranes (Nagy 2022). This process influences the subcellular localization, membrane insertion, and assembly of the viral replication complex (VRC). Silencing HSP70 expression in *Nicotiana benjamina* markedly attenuates TBSV-induced symptoms (Molho et al. 2021). Similarly, during abutilon mosaic virus (AbMV; *Begomovirus bauri*) infection, HSP70 forms a complex with viral movement protein (MP), which accumulates at the cell periphery and in chloroplasts (Kleinow et al. 2020). The downregulation of HSP70 expression in plants inhibits viral cell-to-cell movement but has no significant effect on viral replication (Kleinow et al. 2020). Translationally controlled tumor protein (TCTP) is a highly conserved and ubiquitously expressed protein across eukaryotic species. TCTP interacts with viral proteins and is proposed to support viral replication by stabilizing replication complexes or modulating host translational capacity. The silencing of TCTP expression in tomato or *Nicotiana benthamiana* significantly suppresses pepper yellow mosaic virus (PepYMV; *Potyvirus capsiflavi*) and TuMV infection, respectively (Bruckner et al. 2022, 2017). Moreover, the silencing of TCTP in *Nicotiana tabacum* leads to an almost complete absence of symptoms upon potato virus Y (PVY) infection (Guo et al. 2017). Taken together, these results emphasize that plant viruses hijack conserved host proteins to sustain efficient replication and promote disease development.

### Role of protein interactions in the activation of the host defense mechanism

Viruses modulate plant cells and their defense systems to promote infections. As plants and viruses have coevolved, they have developed various resistance mechanisms for survival that synergistically increase defense against viral infections. Broadly, these strategies operate through two complementary layers. On the one hand, plants can actively perceive viral invasion and initiate immune responses through specific protein–protein interactions. On the other hand, plants can restrict viral accumulation or intercellular movement, thereby conferring resistance or tolerance that often results in attenuated disease symptoms compared with susceptible plants (Garcia-Ruiz 2019). For instance, a RING1-IBR-RING2–type E3 ubiquitin ligase, RBRL, has been shown to recognize the coat proteins of both Rice stripe virus (RSV; *Tenuivirus oryzaclavatae*) and RDV, thereby functioning as a viral sensor. Viral perception enhances RBRL activity, leading to the ubiquitination and degradation of the JA signaling repressor NINJA3 and the activation of JA-mediated antiviral immunity (Huang et al. 2025). In addition to such sensor-like mechanisms, plants also deploy classical resistance proteins to recognize viral components and restrict viral spread. The Tm-22 gene in tomato encodes a CC-NBS-LRR protein that recognizes the C-terminus of the movement protein (MP) of tomato mosaic virus (ToMV; *Tobamovirus tomatotessellati*) (Weber and Pfitzner 1998), which triggers a robust antiviral response in host plants, conferring enhanced antiviral resistance. Simultaneously, viral mutants lacking the C-terminal segment of MP can overcome the resistance imparted by the Tm-22 gene in tomato, enabling its translocation within the foliar tissues of tobacco. Similarly, the tomato immune receptor protein Sw-5b confers broad-spectrum resistance to tospoviruses by recognizing a highly conserved peptide composed of 21 amino acids (NSm21) within the MP NSm encoded by the virus (Zhu et al. 2017).

Interestingly, the virus can overcome Sw-5 resistance when the 118th amino acid of the TSWV MP is changed from cysteine (C) to tyrosine (Y) (C118Y) or when the 120th amino acid is changed from threonine (T) to aspartic acid (N) (T120N). Therefore, the tomato Sw-5 protein interacts with the TSWV movement protein, triggering a resistance response (Huang et al. 2022). The restricted-TEV-movement (RTM) genes RTM1, RTM2, and RTM3 are expressed in phloem sieve elements, which interact with the viral CP to restrict the systemic movement of tobacco etch virus (TEV; *Potyvirus nicotianainsculpentis*), as well as certain isolates of plum pox virus (PPV; *Potyvirus plumpoxi*) (Chisholm et al. 2001). During plant virus‒host interactions, some host kinases also play important roles by directly phosphorylating viral proteins, thereby suppressing viral infection. cAMP-dependent protein kinase (PKA) can phosphorylate the BBSV CP at threonine 41 (T41) (Zhao et al. 2015) and the BSMV γb protein at Ser96 in vivo and in vitro (Zhang et al. 2020a, b, c). The triple gene block 1 (TGB1) protein of BSMV is essential for viral intercellular movement. The protein kinase CK2 can phosphorylate TGB1 (Hu et al. 2015). However, replacing either Thr-395 or Thr-401 with aspartic acid disrupts intercellular movement across monocot and dicot plants, preventing systemic infections.

In host cells, viruses can induce the remodeling of internal membranes or organelles, such as the endoplasmic reticulum and peroxisomes, and the formation of specific cellular structures, such as spherules, vesicles, and multivesicular bodies, which encapsulate viral replication complexes. Plants deploy various defense strategies against viral infections, including the use of decoy proteins that bind with viral proteins, thereby obstructing their ability to identify crucial host proteins associated with translation and replication systems. Moreover, plants can phosphorylate viral proteins, blocking viral infections. Collectively, these defense mechanisms target viral components.

These reports collectively validate that in the coevolutionary arms race between plants and viruses, both parties can develop their own “weapons”, which involve various mechanisms underlying plant antiviral and viral counterdefense.

## Knowledge of virus‒host protein interactions helps develop crop protection strategies

Viruses constitute a group of the most important plant pathogens that cause serious crop losses worldwide. Agricultural statistics reveal that viral infection-associated annual yield losses exceed $30 billion (Jones 2021). In contrast to crop diseases, plant viral infections cannot be eradicated using pesticides, and highly resistant germplasms are scarce. Investigating viral pathogenesis and host resistance is crucial for the development of new strategies to effectively control disease progression. Identifying key antiviral genes for breeding can significantly contribute to sustainable disease management and enhance agricultural sustainability and food security.

### Construction of virus‒host protein interaction network maps

Recent advancements in genomic technologies have generated large amounts of data on protein interactions at an unprecedented pace. Interactions between proteins form intricate networks within cells. By analyzing these networks, we can predict the functions of proteins on the basis of their positions within cellular interaction networks. GPIBase (Wang et al. 2023), a geminivirus-specific database, integrates diverse datasets to provide a valuable resource containing genetic information on *geminiviruses*, their hosts, vectors, and the associated interactions among viruses, plants, and insects. Moreover, this database facilitates the rapid identification of molecules strongly associated with geminivirus infection, including both viral targets and host factors that play critical roles during infection. It also supports the discovery of host proteins closely associated with viral proteins, thereby providing valuable candidates for dissecting the molecular basis of geminivirus dependence and pathogenesis.

While interaction network maps mainly reveal which proteins may interact, the structural details of these interactions, as well as the specific residues and interfaces involved, often remain elusive. In this regard, AI-assisted structural prediction tools, such as AlphaFold and AlphaFold-Multimer, offer a powerful complement by enabling the identification of interaction interfaces, hotspot residues, and candidate targets for resistance engineering. For example, AlphaFold-Multimer has been shown to predict cross-kingdom interactions at the plant–pathogen interface, demonstrating its value in uncovering candidate interaction complexes (Homma et al. 2023). In addition, AlphaFold-guided redesign of a plant pectin methylesterase inhibitor has already enabled the rational improvement of broad-spectrum disease resistance, highlighting the translational potential of structure-guided engineering (Xia et al. 2024). Together, these studies indicate that integrating interaction network maps with AI-assisted structural prediction will greatly enhance our ability to decode virus–host interaction mechanisms and accelerate the rational design of disease-resistant crops.

Despite this progress, the development of plant virus protein databases and interaction resources remains far less mature than that of comparable databases for human and animal viruses. Moreover, virus–host protein interaction networks are inherently dynamic and continuously reshaped during infection. In response to viral invasion, plants alter the abundance of numerous defense- and stress-related proteins, while the associated PPIs vary across tissues, developmental stages, and infection time points. This spatiotemporal plasticity creates an ever-changing interaction landscape, making it difficult to construct network maps that fully capture the complexity of plant–virus interactions. Therefore, continued and systematic efforts will be required to build more comprehensive and dynamic PPI resources, which are essential for improving the precision and practical value of future analyses aimed at dissecting virus–host interaction mechanisms.

### Strategies for disrupting critical protein‒protein interactions to impede viral replication and spread

Studies of virus-host PPIs provide a rational framework for antiviral molecular breeding. Systematic analysis of these interaction networks enables the identification of two major classes of genetic targets-susceptibility (S) genes, which encode host proteins hijacked by viruses to facilitate replication or movement, and resistance (R) genes, which encode proteins that mediate immune recognition and defense activation. On the basis of these interaction maps, antiviral breeding strategies can employ targeted genome editing to knock out or knock down S gene expression, thereby disrupting viral infection pathways, while overexpressing R genes to activate host defense responses. This integrative approach facilitates the development of crop varieties with durable and potentially broad-spectrum resistance to viral.

**Fig. 3:**
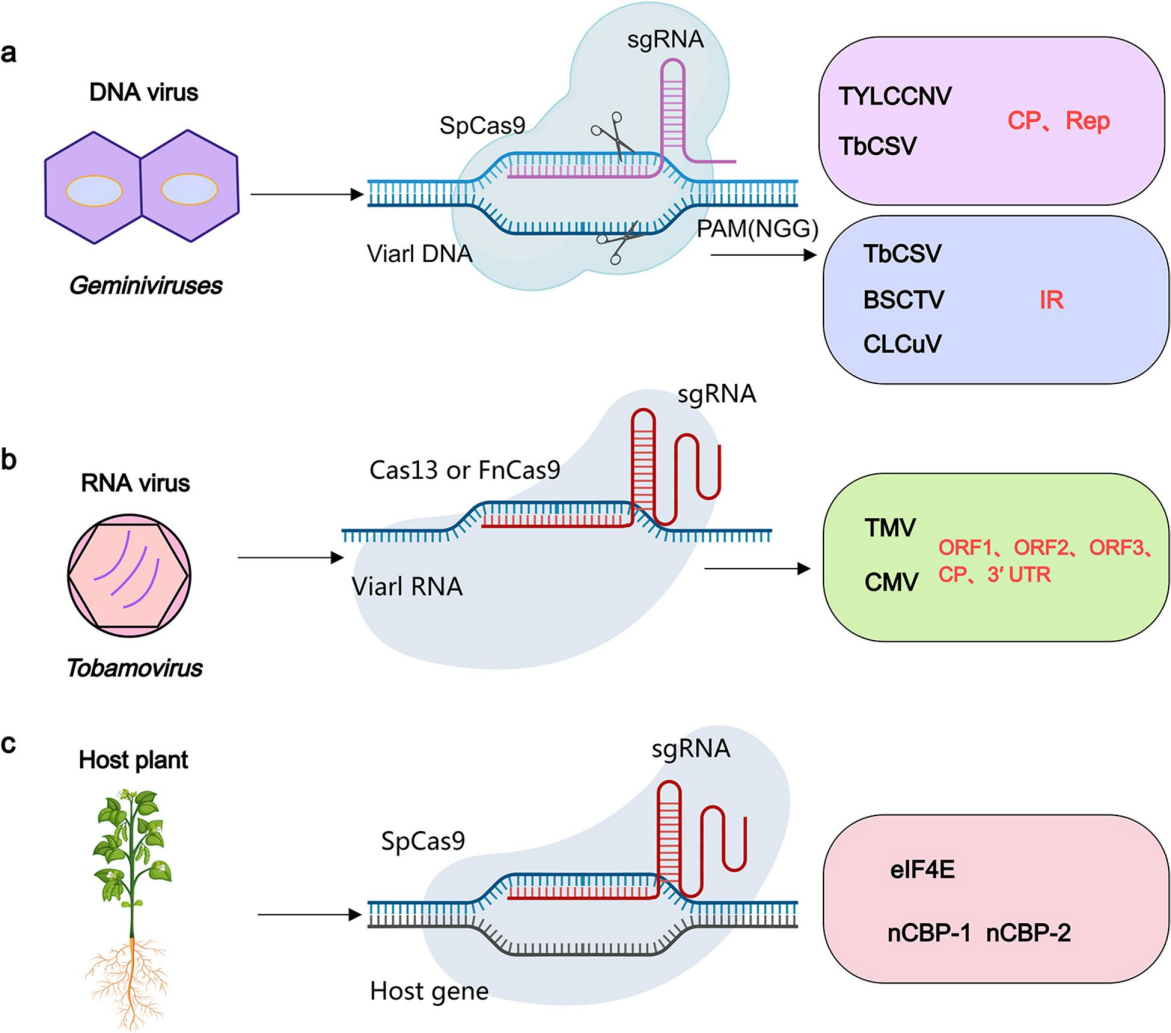
Schematic Diagram of CRISPR/Cas Strategies Targeting Plant Viruses and Host Factors. **a** CRISPR/Cas System Targets Plant DNA Virus Genomes. *Geminiviruses*, as representatives of DNA viruses, can be targeted and cleaved by sgRNA of the Cas9 protein. The sgRNA targets the CP and Rep coding regions or the intergenic region IR of the virus Genomes. **b** CRISPR/Cas System Targets Plant RNA Virus Genomes. TMV and CMV as representatives of RNA viruses, can be targeted and cleaved by sgRNA of the Cas13 protein or FnCas9. The sgRNA targets the ORF1, ORF2, ORF3, CP and 3’UTR of the virus Genomes. **c** CRISPR/Cas System Targets Plant host Genomes. Eukaryotic translation initiation factors eIF4E and novel cap-binding protein-1 (nCBP-1) and nCBP-2 can be edited to potentially confer resistance to virus infection or to alter plant responses. The abbreviations of the virus names in this figure are as follows: TYLCSV, tomato yellow leaf curl Sardinia virus; TYLCV, tomato yellow leaf curl virus; TMV, tobacco mosaic virus; CMV, cucumber mosaic virus; BSCTV, Beet Severe Curly Top Virus; CLCuV, the Cotton Leaf Curl Virus

In recent years, clustered regularly interspaced short palindromic repeat (CRISPR)/CRISPR-associated (Cas) systems derived from bacteria and Archaea have been incorporated into research on viral resistance in plants. These systems represent pivotal adaptive immune defense systems against invasive nucleic acids and have significant potential in plant antiviral breeding studies (Zhao et al. 2020). To promote viral resistance in plants, CRISPR/Cas technology is used via two principal strategies. The first strategy involves the precise targeting and disruption of the viral genome to significantly impede viral replication and subsequent invasion. The second strategy focuses on the nuanced modulation of host factors that are pivotal for viral infection or the life cycle; this method increases plant immunity and serves as a deterrent to viral infections, thereby offering a dual-pronged defense mechanism against plant pathogens (Fig. 3) (Pyott et al. 2016; Shan-E-Ali et al. 2016).

Editing host susceptibility gene through CRISPR/Cas-mediated genome editing has proven to be an effective strategy to reduce viral replication and accumulation. For example, cassava encodes five members of the eIF4E family. Among these proteins, only nCBP-1 and nCBP-2 interact with the VPg protein of the cassava brown streak virus (CBSV; *Ipomovirus brunusmanihotis*), which greatly affects cassava production (Gomez et al. 2019). Targeted disruption of such virus-dependent host factors through CRISPR/Cas-mediated genome editing can significantly reduce viral replication and accumulation, thereby mitigating disease severity and yield loss. Beyond simple knockout of host susceptibility genes, recent work highlights the potential of precision editing at virus-targeted host interaction interfaces. In rice, the RGSV P3 protein suppresses strigolactone-mediated antiviral defense by binding the SL receptor D14. Structural analysis identified D14 residue D102 as essential for P3 binding but dispensable for SL perception, and cytosine base editing of this site (D102N) abolished the P3–D14 interaction while preserving plant growth and yield, thereby conferring strong resistance to RGSV in both japonica and indica rice backgrounds (Yang et al. 2026).

In addition to editing host factors, CRISPR/Cas systems targeting viral DNA or RNA genomes have been successfully applied to enhance resistance against diverse plant viruses. These proteins execute their functions by cleaving pivotal sequences within the viral genome, and disrupt fundamental viral processes, including infection, replication, and transcription (Zainul et al. 2022). For example, expression of Cas9/sgRNA in *Nicotiana benthamiana* confers effective resistance to several *geminiviruses*, including tomato yellow leaf curl China virus (TYLCCNV; *Begomovirus solanumflavuschinaense*) and tobacco curly shoot virus (TbCSV; *Begomovirus nicotianae*), by targeting regions critical for viral replication (Cheng et al. 2015) (Chen et al. 2014). Furthermore, CRISPR/Cas9 systems employing sgRNAs specifically targeting either the viral replication sites or the intergenic regions (IR) more effectively limit viral infections than the CP or Rep-specific sgRNAs. This approach has successfully enhanced the resistance in transgenic *N. benthamiana* against viruses, such as the beet severe curly top virus (BSCTV; *Curtovirus betae*) and the cotton leaf curl virus (CLCuV; *Begomovirus gossypii*) (Balts et al. 2015; Ji et al. 2015). Recently, Cas9 from *Francisella novicida* (FnCas9) and Cas13a from *Leptotrichia shahii* (LshCas13a) were reported to effectively cut RNA strands (Cao et al. 2020). Based on the CRISPR/FnCas9 system, researchers have developed sgRNAs targeting the genomes of the cucumber mosaic virus (CMV; *Cucumovirus CMV*) and TMV, which has led to the establishment of genetically stable *N. benthamiana* and *Arabidopsis* plants, with offspring showing considerably fewer symptoms of viral infection and significantly suppressed viral replication (Zhang et al. 2018).

In parallel with gene editing or identifying favorable natural variation sites in S genes such as *RBL1* and *ROD1* in rice and *MLO* in wheat, which can enhance disease resistance without compromising yield traits, enhancing host resistance through R genes represents a complementary breeding strategy (Gao et al. 2024). Plants have evolved R-genes that mediate their resistance to a wide range of pathogens, including viruses. R proteins recognize viral components either directly or indirectly and activate downstream immune signaling pathways. Notably, the N gene is a key R gene that has been extensively explored owing to its role in signal transduction against TMV; it belongs to the TIR-NBS-LRR class of viral resistance genes and functions by indirectly interacting with the TMV helicase through its N protein, which triggers a hypersensitive response and viral resistance, effectively containing TMV at the initial infection site (Abbink et al. 1998). In addition, the TIR domain and nuclear localization of the N protein are essential for antiviral resistance, underscoring the importance of spatial regulation of R-protein function (Burch-Smith et al. 2007). It should be noted that resistance mediated by a single R gene is often highly specific to particular pathogen strains. However, some NLR-type R genes found in crops are tightly linked in the genome, forming gene clusters (Meyers et al. 2003). Improving crop disease resistance by combining multiple efficient R genes with complementary resistance spectra is a viable strategy. In wheat, the aggregation of the two monogenic stripe rust resistance genes Yr5 with Yr15 or Yr64 with Yr15 resulted in complete resistance to all tested stripe rust races (Klymiuk et al. 2018). Similarly, different combinations of powdery mildew resistance genes Pm2, Pm4a, and Pm21 have been successfully integrated into the wheat cultivar ‘Yang 158’, conferring broad-spectrum resistance to powdery mildew (Qu et al. 2020).

Overall, the CRISPR/Cas system-based editing of host susceptibility factors or molecular breeding to increase the expression of host resistance genes can significantly increase plant defense against viruses. However, single-point mutations generated by the CRISPR/Cas system at genomic target sites may induce rapid adaptation in viruses, leading to the emergence of new variants. Therefore, improvements in the genome-editing efficacy of the CRISPR/Cas9 system is urgently needed to overcome viral evasion strategies through point mutations, thereby minimizing the risk of diminished or impaired resistance to viral infections.

## Summary and prospects

**Fig. 4:**
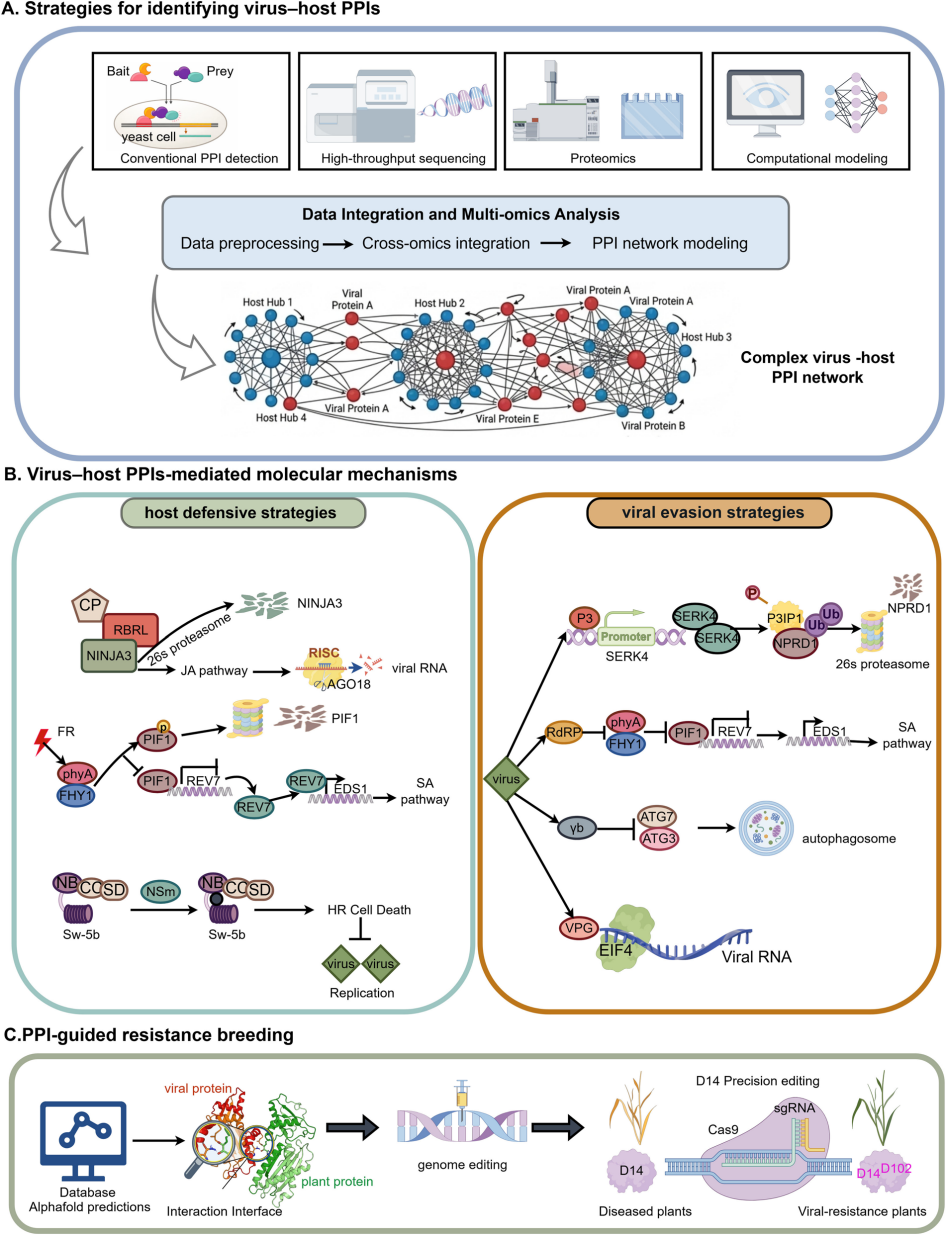
Conceptual integrative model of the PPIs in plant‒virus interactions. **a** Strategies for identifying virus–host PPIs. Conventional PPI detection methods, high-throughput sequencing, proteomics, and computational modeling collectively generate multidimensional datasets. these datasets can be integrated to construct complex virus–host PPI networks. **b** Representative molecular mechanisms mediated by virus–host PPIs. On the left, hosts deploy defense pathways such as hormone signaling, RNA silencing, and HR-associated immunity. On the right, viruses escape host defense by targeting key immune regulators, suppressing antiviral signaling pathways, hijacking the host translational machinery, and disrupting autophagy- or proteasome-mediated degradation pathways. **c** Insights from PPI networks and structural interfaces can be translated into resistance breeding through precision genome editing of key host or viral interaction determinants

Given that these interactions are inherently transient, low-affinity and highly dynamic, no single method is sufficient to fully resolve the complexity of the interactome. Thus, high-throughput sequencing, proteomics and computational modelling should be integrated as complementary approaches. In particular, AI-based structural prediction tools, such as AlphaFold-Multimer, have emerged as powerful complements to experimental methods by enabling the systematic modeling of virus–host interaction interfaces and facilitating the prioritization of candidate interactions for validation. Notably, the combined application of affinity purification–mass spectrometry (AP–MS), proximity-labelling techniques, structural prediction and network analysis is driving a transition from the identification of individual protein pairs to system-level characterization of virus–host interaction networks. Nevertheless, the investigation of plant virus‒host interaction networks is still in its infancy, and the paucity of data significantly limits the construction of complex network maps, highlighting the urgent need for enhanced research and data integration. In the future, the integration of high-throughput datasets with multi-omics frameworks and quantitative MS platforms, coupled with AI-driven structural and interaction predictions, will further improve the spatiotemporal resolution of virus–host PPI analysis and deepen our understanding of the dynamics and functional significance of virus–host interactions (Fig. 4a).

A comprehensive dissection of virus–host PPI networks reveals a continuously evolving molecular “arms race” between host defense and viral counter-defense strategies (Fig. 4b). For host defensive strategies, plants have evolved multilayered immune systems, in which PPIs serve as central regulatory hubs that enable the perception of viral invasion and the activation of diverse defense pathways, including RNA silencing, phytohormone signaling, MAPK cascades, autophagy, and hypersensitive cell death (Gong et al. 2025; Huang et al. 2025; Khan et al. 2008; Zhu et al. 2017). For viral evasion strategies, viruses have evolved sophisticated counter-defense strategies, such as encoding potent RNA silencing suppressors, reprogramming host factors, and hijacking host degradation systems, thereby evading or suppressing host immune surveillance to promote viral replication and systemic infection (Wu et al. 2025; Yang et al. 2018; Zhang et al. 2020a, b, c; Zou et al. 2025). Collectively, these findings highlight that key node within virus–host PPI networks function as critical regulatory hubs controlling the outcome of viral infection.

The value of virus–host PPI research extends well beyond mechanistic understanding and is increasingly informing antiviral crop improvement. The identification of hub proteins, critical interaction interfaces, and key susceptibility or resistance determinants provides a rational foundation for precision breeding (Yang et al. 2026). In particular, integrating PPI network analysis with AlphaFold-based structural prediction and CRISPR/Cas-mediated genome editing opens new opportunities for engineering virus resistance in a targeted manner. Such strategies can be used not only to directly disrupt viral genomes, but also to modify host factors or interaction interfaces that are required for viral invasion, replication, or spread. Compared with traditional breeding, this PPI-guided approach offers greater precision and holds strong potential for developing broad-spectrum and durable resistance (Fig. 4c).

In conclusion, elucidating the complex interactions between plant and viral proteins is essential for understanding how plants coordinate antiviral immunity with broader cellular stress responses. The continued integration of interactome mapping, AI-assisted structural prediction, and functional validation will not only deepen mechanistic insights into plant–virus interactions but also establish a robust foundation for the rational design of antiviral strategies and the breeding of virus-resistant crops.

## Data Availability

All data and material generated or analyzed during this study are included in this published article.

## Abbreviations

ABA: Abscisic acid
AbMV: Abutilon mosaic virus
AP-MS: Affinity purification coupled with mass spectrometry
BBSV: Beet black scorch virus
BiFC: Bimolecular fluorescence complementation
BSCTV: Beet severe curly top virus
BSMV: Barley stripe mosaic virus
CBSV: Cassava brown streak virus
CF-MS: Cofractionation mass spectrometry
CMV: Cucumber mosaic virus
Co-IP: Co-Immunoprecipitation
CP: Coat protein
FRET: Fluorescence resonance energy transfer
IR: Intergenic regions
ITC: Isothermal titration calorimetry
JA: Jasmonic acid
MAPK: Mitogen-activated protein kinase
MP: Movement protein
MS: Mass spectrometry
MST: Microscale thermophoresis
PL: Proximity labeling
PPI: Protein-protein interaction
PPI: Protein-protein interaction
PPV: Plum pox virus
RDV: Rice dwarf virus
RdDM: RNA-directed DNA methylation
RdRPs: RNA Dependent RNA Polymerases
RGSV: Rice grassy stunt disease
RSV: Ricestripevirus
SA: Salicylic acid
TbCSV: Tobacco curly shoot virus
TCV: Turnip crinkle virus
TEV: Tobacco etch virus
TMV: Tobacco mosaic virus
ToMV: Tomato mosaic virus
TuMV: Turnip mosaic virus
TYLCCNV: Tomato yellow leaf curl virus
VSR: Viral suppressors of RNA silencing
